# Heating Pad–Induced Burns Leading to Rhabdomyolysis and Acute Kidney Injury: Case Report

**DOI:** 10.7759/cureus.107525

**Published:** 2026-04-22

**Authors:** Meghna Kurup, Swet Patel, James Espinosa, Alan Lucerna

**Affiliations:** 1 Emergency Medicine, Jefferson Health NJ, Stratford, USA

**Keywords:** acute kidney injury, burns, contact burn, heating pad, : rhabdomyolysis, thermal injury

## Abstract

Heating pads are commonly used for pain relief and are generally considered safe; however, prolonged or inappropriate use may result in significant thermal injury. We report the case of a 26-year-old male with a history of psychiatric illness and substance use who presented to the emergency department with deep partial-thickness burns to the back following prolonged heating pad use. Laboratory evaluation revealed severe rhabdomyolysis (creatine kinase 16,823 IU/L) and acute kidney injury (creatinine 11.56 mg/dL), necessitating transfer to a burn center and initiation of renal replacement therapy.

Despite involving approximately 10% total body surface area, the burn resulted in significant systemic complications. The patient improved with aggressive fluid resuscitation, dialysis, and surgical management, including grafting, and was discharged after recovery.

This case highlights that relatively limited burns from low-intensity heat sources can result in deep tissue injury, rhabdomyolysis, and severe acute kidney injury. Early recognition and prompt management are critical to improving outcomes.

## Introduction

Thermal injuries from heating pads are uncommon but well-documented, typically presenting as superficial burns following chronic exposure [1]. These injuries are often underestimated in terms of depth and severity [2]. In particular, low-intensity heat sources may result in insidious injury due to prolonged exposure, which can delay recognition and increase the risk of deeper tissue involvement.

Rhabdomyolysis and acute kidney injury (AKI) are well-recognized complications of severe burns, particularly when total body surface area (TBSA) involvement exceeds 20% [3]. Acute kidney injury is associated with increased morbidity and mortality in burn patients [4]. However, severe rhabdomyolysis and dialysis-requiring AKI can also occur in the setting of relatively limited TBSA involvement, although this is less well characterized.

We present a case of heating pad-induced burns involving approximately 10% TBSA complicated by severe rhabdomyolysis and AKI requiring dialysis, highlighting the potential for significant systemic injury even with localized thermal exposure. This case highlights an underrecognized mechanism of injury and demonstrates the importance of early recognition and management.

## Case presentation

A 26-year-old male with a history of schizophrenia and opioid use disorder presented to the emergency department with painful burns to his back after using an electric heating pad for several days before presentation. The patient was unable to specify the duration or pattern of use. 

**Figure 1:**
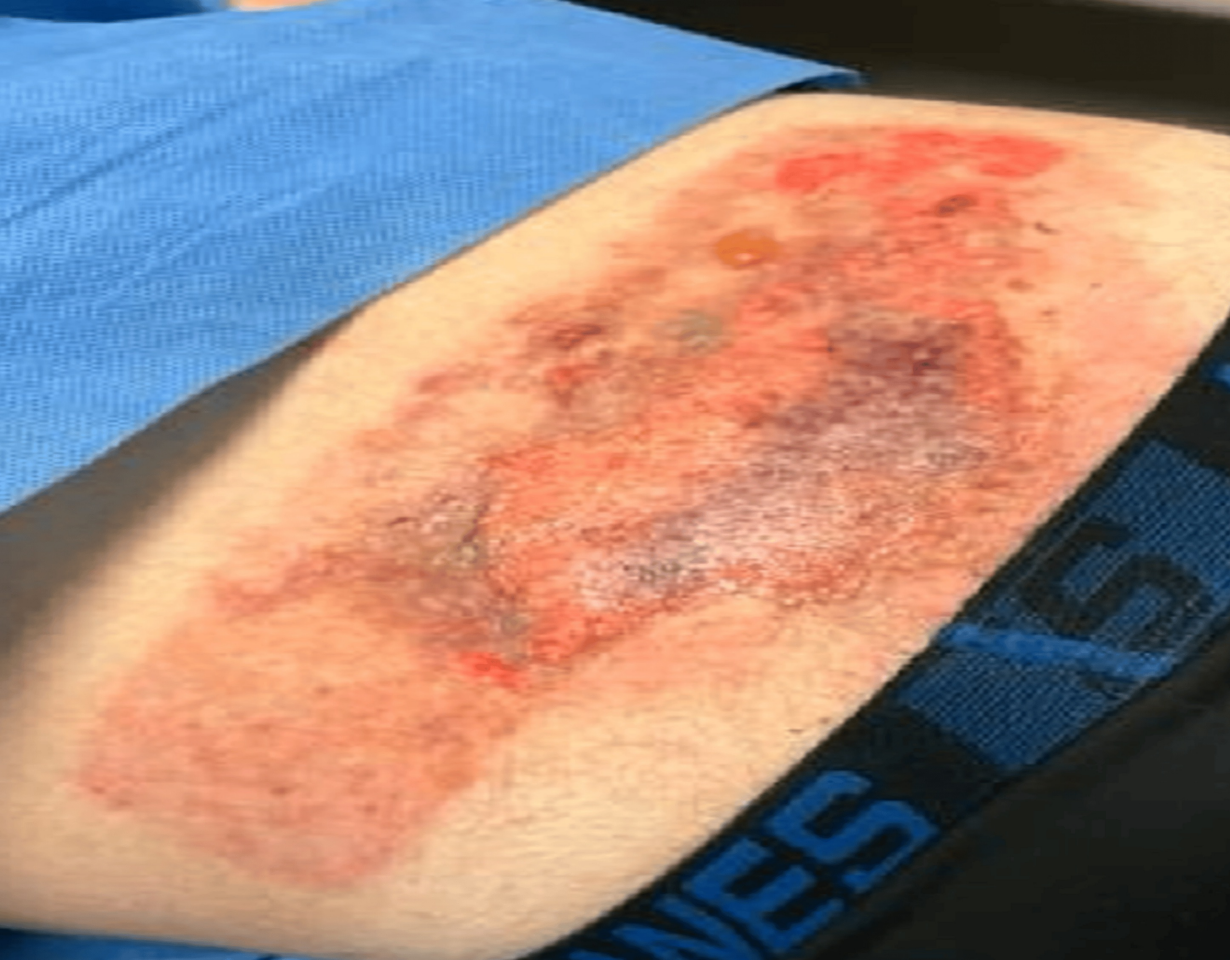
Deep partial-thickness burn over the upper back (~20 × 20 cm) with erythema and blistering.

**Figure 2:**
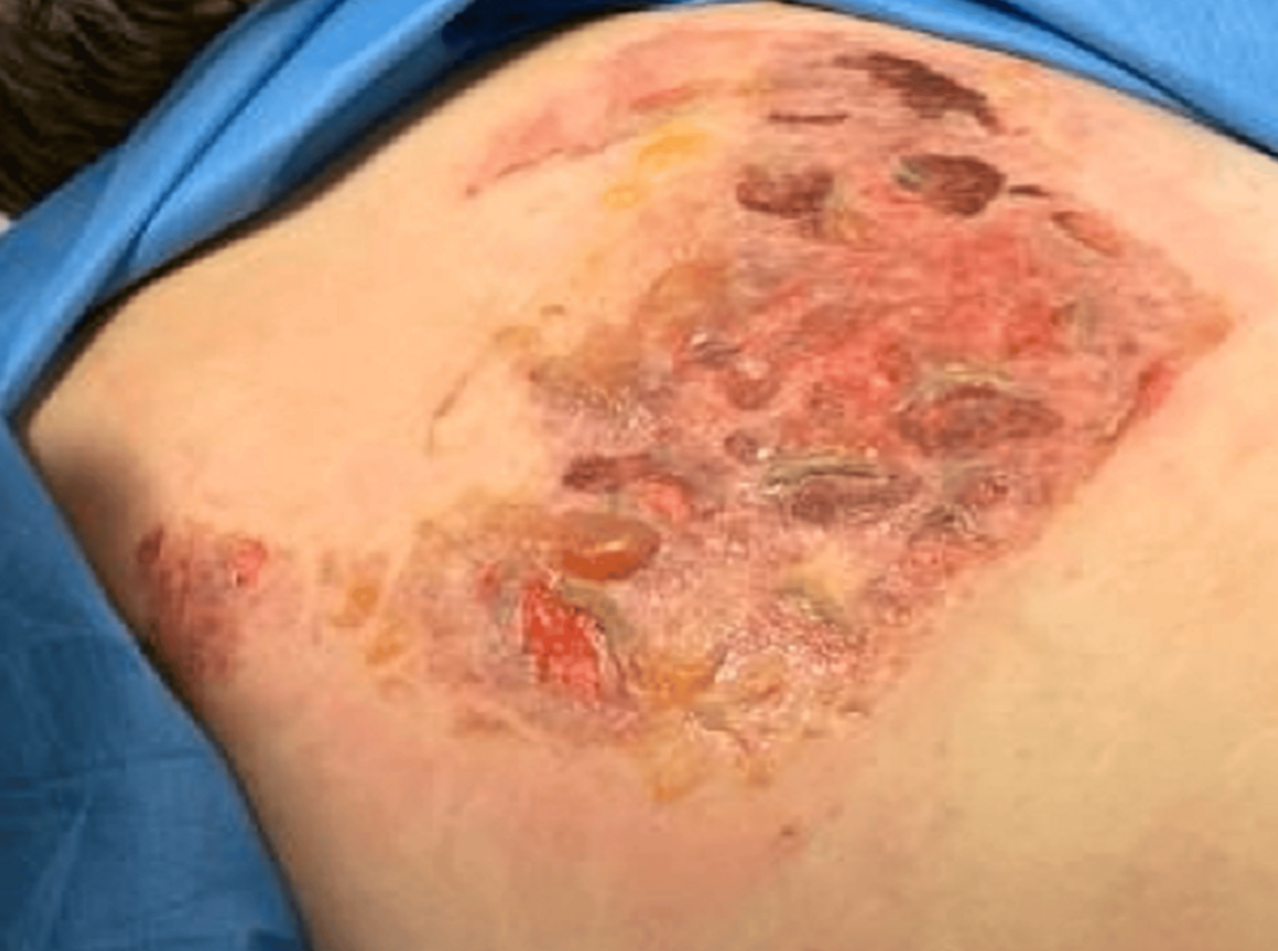
Deep partial-thickness burn over the lower back (~20 × 10 cm) with erythema, blistering, and areas of charring, demonstrating a localized contact pattern.

On arrival, vital signs showed a heart rate of 80 beats per minute, a respiratory rate of 36 breaths per minute, a blood pressure of 141/96 mmHg, and a temperature of 99°F. Physical examination revealed two areas of deep partial-thickness burns: a 20 × 20 cm area over the upper back (Figure 1) and a 20 × 10 cm area over the lower back (Figure 2), both with erythema, blistering, and areas of charring. Total burn surface area was estimated at approximately 10%.

Initial laboratory evaluation demonstrated severe rhabdomyolysis, with a creatine kinase level of 16,823 IU/L, and acute kidney injury, with a creatinine of 11.56 mg/dL (Table 1). Blood cultures obtained prior to the initiation of antimicrobial therapy showed no growth. 

**Table 1:** Laboratory Results. BUN: blood urea nitrogen; PT: prothrombin time; PTT: partial thromboplastin time; CK: creatine kinase, INR: international normalized ratio; AST: aspartate aminotransferase; ALT: alanine aminotransferase; K/uL: thousands per microliter; g/dL: grams per deciliter; mEq/L: milliequivalents per liter; mg/dL: milligrams per deciliter; mmol/L: millimoles per liter; IU/L: international units per liter; mm3: cubic millimeter; cells/HPF: cells per high-power field.

Laboratory Results	Result	Normal Range	Units
White blood cell count	6,000	4.0-11.0	mm3
Hemoglobin	12.2	10.6-15.6	g/dL
Platelet count	190	150-400	K/uL
Sodium	136	135-154	mEq/L
Potassium	4.0	3.5-5	mEq/L
BUN	32.0	5-20	mg/dL
Creatinine	11.6	0.6-1.2	mg/dL
CK	16823.0	22-198	IU/L
Glucose	85.0	70-100	mg/dL
Calcium	8.9	8.5-10.5	mg/dL
Chloride	103.0	95-105	mEq/L
Bicarbonate	25.0	23-29	mEq/L
Magnesium	1.9	1.7-2.2	mg/dL
Lactate	1.5	0.5-2.2	mmol/L
PT	11.0	11-13.5	sec
PTT	33.0	25-35	sec
INR	1.0	0.8-1.1	INR ratio
Total bilirubin	1.1	0.1-1.2	mg/dL
Direct bilirubin	0.3	0-0.3	mg/dL
AST	30	8-33	IU/L
ALT	52	7-56	IU/L
Alkaline phosphatase	183	39-117	IU/L
Urine color	clear	yellow	NA
Urine clarity	clear	clear	NA
Urine specific gravity	1.0	1.005-1.030	NA
Urine pH	7.0	5-7.5	NA
Urine glucose	negative	negative	NA
Urine protein	negative	negative	NA
Urine bilirubin	positive	negative	NA
Urine urobilinogen	positive	negative	NA
Urine ketones	negative	negative	NA
Urine blood	negative	negative	NA
Urine white cells	negative	0-5/HPF	cells/HPF
Urine red cells	negative	0-5/HPF	cells/HPF
Urine nitrite	negative	negative	NA
Urine leukocyte esterase	negative	negative	NA
Blood culture	no growth	no growth	NA

Fluid resuscitation was initiated with lactated Ringer’s solution guided by the Parkland formula (approximately 3,160 mL over the first 24 hours based on estimated body weight and burn surface area). Antibiotics were not initiated, as there was no clinical indication for antimicrobial therapy. Pain was managed with intravenous opioids.

Given the severity of the injury, the patient was transferred to a regional burn center for further management.

During hospitalization, the patient required hemodialysis for acute kidney injury and subsequently underwent surgical debridement and skin grafting. Renal function gradually improved, with creatinine decreasing to 3.07 mg/dL and creatine kinase declining to 118 IU/L.

Psychiatric evaluation determined that the injury was unintentional. The patient was discharged on hospital day 21 with resolution of acute kidney injury and was advised to continue follow-up with his outpatient psychiatric providers; however, outpatient follow-up information was not available.

## Discussion

Rhabdomyolysis and acute kidney injury are well-recognized complications of thermal injury and may result from direct muscle damage and systemic inflammatory responses.

This case demonstrates that prolonged exposure to a low-intensity heat source can result in deep tissue injury with significant systemic consequences. Although heating pad-related injuries are often considered low risk, prolonged localized exposure may lead to unrecognized muscle damage beneath areas of partial-thickness burns.

Rhabdomyolysis in burn patients results from direct muscle injury, leading to the release of intracellular contents, including myoglobin, creatine kinase, phosphate, and urate into the circulation [3]. Myoglobin plays a central role in the development of AKI by precipitating within renal tubules, causing obstruction, oxidative injury, and reduced glomerular filtration [3]. In this case, prolonged contact likely resulted in deeper tissue injury than was clinically apparent, leading to skeletal muscle necrosis.

Rhabdomyolysis is more commonly associated with burns involving greater than 20% total body surface area (TBSA). In contrast, this patient developed severe rhabdomyolysis and dialysis-requiring AKI with approximately 10% TBSA involvement, suggesting that duration and intensity of exposure may be as important as surface area in determining systemic risk.

AKI in burn patients is multifactorial, involving myoglobin-mediated tubular injury, hypovolemia from fluid shifts, evaporative losses, and systemic inflammation [4]. Early AKI is often related to hypovolemia, whereas later AKI is more commonly associated with infection and multi-organ dysfunction [4,5]. AKI is associated with increased morbidity and mortality in burn patients [6,7], and rhabdomyolysis is an independent risk factor for its development [8,9].

Contact burns from therapeutic devices have been described, particularly in patients with impaired sensation or altered mental status [1]. In this case, psychiatric comorbidity and unclear exposure duration likely contributed to prolonged contact and delayed presentation. The geometric distribution of the burns supports a localized contact mechanism.

Diagnosis of rhabdomyolysis is supported by elevated creatine kinase levels, with higher levels correlating with increased risk of AKI and need for renal replacement therapy [10]. Management centers on early recognition and aggressive fluid resuscitation to maintain renal perfusion and facilitate myoglobin clearance. When renal failure develops, timely initiation of renal replacement therapy is essential [11]. This patient’s course, including the need for dialysis followed by recovery, underscores the importance of prompt escalation of care.

While contact burns from heating devices have been reported, cases of isolated heating pad use resulting in severe rhabdomyolysis and dialysis-requiring AKI with limited TBSA involvement are uncommon. This case highlights the potential for significant systemic complications from prolonged low-intensity thermal exposure and reinforces the importance of early evaluation for rhabdomyolysis in similar presentations.

## Conclusions

Heating pad use, though generally considered safe, can result in significant thermal injury and systemic complications when used improperly. Even burns involving a small total body surface area may cause deep tissue injury, rhabdomyolysis, and severe acute kidney injury requiring renal replacement therapy.

Clinicians should maintain a high index of suspicion for rhabdomyolysis in patients with delayed burn presentations, particularly after prolonged heat exposure. Early recognition, prompt laboratory evaluation, aggressive fluid resuscitation, and timely escalation to renal replacement therapy are essential to optimize outcomes.
